# A Large Vesico-Vaginal Fistula, with Everted Bladder into Vaginal Canal

**Published:** 1888-11

**Authors:** K. P. Moore

**Affiliations:** Ga.


### A LARGE VESICO-VAGINAL FISTULA, WITH EVERTED BLADDER INTO VAGINAL CANAL. CONCEPTION REACHING FOUR AND A HALF OR FIVE MONTHS GESTATION—OPERATION, &c.

BY K. P. MOORE, M. D. OF GA.

[READ BEFORE THE MACON MEDICAL SOCIEEY.]

About the 1st of March, 1888, I was called to see Mrs. F., age about forty. Found a large vesico-vaginal fistula reaching entirely across the superior vaginal wall, and making an aperture into the bladder amply large to pass a large turkey egg. The rent had existed a little over six months and the vaginal walls had become indurated and so much retracted as to allow fully two-thirds or more of the bladder to become everted and protude into the vagina, forming a large, scarlet, granulating mass, which would bleed and give excruciating pain upon the slightest manipulation.

I obtained the following history:

Was confined with her first labor in July, 1887. Child was engaged in inferior straight for six to eight hours, and labor exceedingly hard. Up to this time she was not under the care of any physician. Dr. J. G. McCrary was now called in, but about the time he arrived the pains ceased, and though he used all the usual remedies for some hours, he failed to reestablish the labor. He now left and went in search of Dr. Mettauer, who had previously been sent for. About the time that they reached the patient's bed side, the pains again began and she was soon delivered of a large child without the aid of instruments. For a while everything seemed to progress satisfactorily, although she never felt well. She soon made the unwelcome discovery that her urine was dribbling away from her, and was coming out through the vagina. The labia and inner parts of her thighs now became excoriated and very sore; she came to possess the disgusting ammoniacal scent and was now a scource of loathing to herself and her friends. Forced to stay at home, and shut up a prisoner, with only her miserable sufferings as her constant companion, she gradually became a nervous wreck and was almost ready to crave death as a relief from her tortures Just here comes in a remarkable chapter in the history of her case Notwithstanding this terrible state of affairs, with the vagina plugged up, with the dribbling sensitive bladder, yet she believed herself to be pregnant, and in the fourth month of gestation.

The cervix did not protrude into the vagina at all, indeed it was with difficulty that I could find it. It was a mere little depression in the superior vaginal wall about one inch behind the fistula. With the indurated and sensitive condition of the parts I found it impossible to make out even the size or exact position of the uterus, and could not arrive at a solution of the question of pregnancy. Drs. F. Walker and J. G. McCrary were now called in and the whole field fully surveyed.

If we could be certain that pregnancy existed, would it not be wise policy to bring on abortion? Should she go to full term, would not the risk of a large slough of the bladder, in spite of every effort to keep it up, be sufficiently urgent to demand an abortion, outside any questions of operation? These were some of the questions discussed at this conference; and it was decided that it was wise practice to induce the abortion, if pregnancy existed. Accordingly a sound was introduced into the uterus, and to our surprise passed to the depth of six inches or more. It was freely swept around in the uterus and failed to give any positive evidence of pregnancy. Hearing nothing from this exploration, Drs. Walker and McCrary again introduced the sound the next day with the same negative result. In the uncertainty of the situation, after a few days, I urged that we give her and the fœtus, if any, the benefit of the doubt, and proceed to operate. My idea was that if the operation should produce abortion we could but lose the operation, and if it should not cause abortion, then we would have accomplished our purpose and saved the life of the child; besides putting the mother in the best possible condition for subsequent labor.

On the 27th of March, assisted by Dr. F. Walker, and in the presence of Drs. McCrary, E. G. Ferguson and W. C. Gibson, we proceeded to make the operation. The patient was etherized and placed in the exaggerated lithotomy position of Simon, and the usual operation done.

The size of the opening, and the long accustomed prolapsed condition of the bladder rendered it necessary to plug up the bladder with a large sponge while we vivified the edges. The silver wire was used, and eighteen sutures required to close up the fistula properly.

During the operation we used solution bichlor mercury 1.3000 freely as an antiseptic, and the bladder and vagina freely syringed out with 1.5000 solution of the same after the completion of the operation. A Nelaton flexible catheter was now tied in the urethra and patient put to bed. Several times during the operation, which lasted two or three hours, there was decided indication of heart failure. The pulse became very weak and irregular, and whiskey with morphia was freely introduced under the skin.

For some days every indication pointed to a success. The catheter would occasionally slip out and allow the bladder to fill up, and yet there was no leakage. We felt quite hopeful, and yet as time drew its anxious length along our buoyant hopes began to weaken, as day by day the urine began more and more to find its way through the old fistulous opening.

Our disappointment can well be imagined, when on the tenth or eleventh day after the operation we removed the stitches to find that there had been but little union. The bladder at once came down into the vagina and soon become the same old wedge prizing open the gaping wound.

Defeated but not entirely discouraged, we began as rapidly as possible to clear away the debris for a second attack. Our patient was put upon tonics, properly regulated stimulants, and nutritious diet. To this regime she responded finely, and "Richard gave fine promise of soon being himself again."

A fine crop of threatening abscesses from the hypodermics of whiskey presented to us a right formidable array of enemies; these with the recently mutilated parts caused more or less symptomatic fever, but in spite of them our patient was gaining ground rapidly and I was congratulating myself on the encouraging prospect of soon being able to undertake the second operation. On the morning of the 15th of April, I received a telephone message from Dr. McCrary to go in haste to see Mrs. F. I was soon at her bed-side and to my great mortification found that she had aborted some hours before of a five months fœtus, and had had excessive hemorrhage, the placenta was still retained, and inspite every effort of the doctor she was still flooding. She was now almost completely exsanguinated, and had fainted several times. She was almost totally blind, her face was blanched, and her whole body bathed in profuse clammy perspiration. Her stomach was making the last cadaveric throes, and it looked as if death was inevitable.

That ever ready and faithful little friend, the hypodermic syringe, never did better work since its invention than on this memorable morning. Whiskey, morphine and ergotine were freely used. The foot of the bed was elevated almost to an angle of forty-five degrees so as to supply the brain with as much blood as possible; the vagina was thoroughly tamponed, hot bottles and blankets packed around her and she was given a little time to rally. A telephone message was now sent for Dr. Walker, who very kindly and promptly came to reinforce us. By the time Dr. Walker arrived she had rallied somewhat, and it was now deemed safe to undertake the removal of the placenta, as the only permanent relief from the hemorrhage. We placed her in Sims' position and I introduced Loomis' placental forceps, and with very little trouble delivered the placenta. The uterus was well syringed out with a hot solution Bichloride Mercury 1 5000, and the patient put warmly to bed. We had no further hemorrhage, and our patient rallied as kindly as could be expected under all the circumstance.s The general supporting regime was adopted and she made a rapid and satisfactory recovery, notwithstanding a second installment of abscesses from the hypodermics of whiskey.

On the 23d of May, again assisted by Drs. Walker, McCrary and Ferguson we made the second operation. This time she was placed in Sims' position, and the operation done without an anæsthetic. Remembering the unpleasant effects of the ether, and the threatening serious heart failure in the first operation, we were willing to allow her to establish for herself the heroic reputation of undergoing the operation without the anæsthetic. She was given a half grain of morphine hypodermically, and two ounces of whiskey per orem. Notwithstanding the heroism and fortitude displayed by this woman, yet several times during the operation she showed so much evidence of collapse, and the heart became so irregular and feeble in its action that we were compelled to suspend and renew the whiskey and morphia.

In the first operation I followed more closely the plan of Sims, and used great caution not to include any of the vesical mucus membrane, either in vivifying or in the sutures. In this, I rested more upon the opinions of Simon, and was less afraid of taking in the vesical surface. I began paring a half inch upon the sound mucous membrane of the vagina, and beveled as far as could be to the border of the vesical edge. In taking the stitches, I started them in a half inch outside the vivified surface, and came out about one or two lines inside the vesical border. The cervix now protruded well down into the vagina and occupied its normal position. It was about one inch behind the rent and as I included a half inch in beveling, and a half inch of sound mucus surface in the sutures, it brought the cervix very close to the fistula; indeed two of my sutures came out through the cervical canal and two through the walls of the cervix on either side. The paring reached entirely across the vaginal canal from the tuberosity of one ischii to the other, and required twenty sutures to close it. The vagina and bladder were now syringed out as before, and a flexible catheter tied in the urethra. A full pad of iodoform gauze was now placed against the vulva and supported by a T bandage which completed the opertaion, and patient put comfortable to bed. On the 12th day after the operation the sutures were removed, and to our great gratification there remained no "waste away" to tell the sad tale of her former misery.

She soon gained strength and flesh, and delights herself in helping her husband, who is a gardener, in nursing and gathering his luxuriant vegatables.

The points of interest in this case are obvious. First, the unusual occurence of pregnancy in this extensively diseased condition, especially with the cervix occupying such an obscure position and in such close proximity to the constantly flowing bladder. Second, the unusual resistance of the uterus to abortion, and its wonderful toleration, not only to the explorations of its own cavity, but of the first operation with all of its concomitant irritations. Third, the fortitude and heroism displayed by this woman in having this operation done without an anæsthetic; such endurance as this is known only to women.

Query—Did we do right in the course we pursued, and thereby sacrificing the life of the fœtus? Should we have allowed this poor woman to go on enduring the tortures of the damned, and possibly the loss of her life at the end of the term of gestation? She might possibly have gone to full term, and the bladder might have held back in position, and the child been born alive without serious risk to the mother, but to my mind our duty was clear at the time, and I think yet we did the right thing.

This report would be, to me, incomplete, if I did not take occasion to acknowledge my obligations especially to Dr. F. Walker who was a coworker in every effort, and to Drs. J. G. McCrary, E. G. Ferguson and W. C. Gibson, who rendered such valuable aid and counsel.

